# Ethnobotanical studies on rice landraces under on-farm conservation in Xishuangbanna of Yunnan Province, China

**DOI:** 10.1186/s13002-024-00683-y

**Published:** 2024-04-29

**Authors:** Fei Wang, Tao Sun, Shuai Yu, Chunhui Liu, Zhuo Cheng, Jianxin Xia, Longzhi Han

**Affiliations:** 1 Key Laboratory of Ecological Environment in Minority Areas (Minzu University of China), National Ethnic Affairs Commission, Beijing, 100081 China; 2 College of Life and Environmental Sciences, Minzu University of China, Beijing, 100081 China; 3 Institute of Crop Sciences, Chinese Academy of Agricultural Sciences, Beijing, 100081 China; 4 State Key Laboratory of Crop Gene Resources and Breeding, Institute of Crop Sciences, Chinese Academy of Agricultural Sciences, Beijing, 100081 China; 5 Institute of Agricultural Sciences, Xishuangbanna Prefecture, Jinghong, 666100 China; 6 College of Agronomy and Biotechnology, China Agricultural University, Beijing, 100193 China

**Keywords:** Ethnic culture, Glutinous rice, On-farm conservation, Rice landraces, Xishuangbanna region

## Abstract

**Background:**

A complex interaction and mutual influence exists among landscapes, cultures, and landraces, with rice culture being a typical embodiment of this relationship. The conservation of landraces operates alongside preserving traditional practices. The Xishuangbanna region stands out as a hub for the genetic diversity of landraces, boasting rich genetic resources. Despite the diverse rice resources in this region, a comprehensive and systematic study has not been undertaken.

**Methods:**

From October to November 2023, we collected rice landraces under the on-farm conservation in 18 townships including Menghai, Mengla and Jinghong in Xishuangbanna. Employing semi-structured interviews and various methods, we investigated factors influencing the preservation and loss of rice landraces in the region. Statistical analysis was applied to the agronomic traits of collected local rice, encompassing indica or japonica, glutinous or non-glutinous, grain shape, and hull color as second category traits. The second category included quantitative traits like thousand grain weight and grain length. Rice diversity among different regions, traits, and ethnic groups was assessed using the Shannon–Wiener index. Additionally, clustering analysis via the UPGMA method depicted the distribution characteristics of the resources.

**Results:**

A total of 70 rice landraces were collected in the Xishuangbanna region, each exhibiting distinct characteristics. Differences were observed across regions, trait, naming, and ethnic groups. Diversity analysis revealed that Mengla had the highest diversity, followed by Menghai, while Jinghong exhibited the lowest diversity. The second category of traits displayed broader diversity than the first, with the Dai people’s glutinous rice showcasing greater diversity than other ethnic groups. Cluster analysis categorized the 70 samples into seven groups at a genetic distance of 1.15. Ethnobotanical interviews emphasized the rapid loss of rice landraces resources in Xishuangbanna, with indigenous ethnic cultures playing a vital role in the conservation of rice landraces. Dai traditions, in particular, played a crucial role in protecting glutinous rice resources, showcasing a mutual dependence between Dai culture and glutinous rice.

**Conclusions:**

The rich natural environment and diverse ethnic cultures in Xishuangbanna have given rise to various rice landraces. The Dai, primary cultivators of glutinous rice with higher diversity, intertwine their traditional ethnic culture with the conservation of glutinous rice resources. At the same time, the preserving glutinous rice resources promotes the inheritance of Dai ethnic culture. However, rice landraces are facing the risk of loss. Hence, collecting and documenting rice landraces is crucial. Encourage local communities to sustain and expand their cultivation, promoting on-farm conservation. These measures contribute valuable germplasm and genes for rice breeding and serve as a means of cultural preservation.

## Background

Landscape, culture, and landraces exhibit complex interactions and mutual influence, constituting a specific region's unique social and cultural system, reflecting the close connection and mutual influence between humanity and the environment [1, 2]. Language is an important component of culture diversity [3]. Rice cultivation culture is a typical manifestation of the interaction and influence among landscape, culture, and landraces [4]. For example, the Manggarai region is a seasonally dry agroclimatic zone with high solar intensity and significant wind. With low rainfall, it mainly cultivates upland rice and has many unique traditional rituals associated with upland rice varieties. The breeding and domestication of these upland rice varieties conform to local cultural customs and environmental constraints [5].

The landscape is an essential foundation for the formation and development of indigenous cultures and landraces [6, 7]. Different landscape and climate conditions influence local residents' language, cuisine, customs, and religious beliefs, thus shaping unique regional cultures [8, 9]. For example, due to their long-term residence along the Dulong River with its abundant natural resources and isolated transportation, the Dulong people have developed a longstanding culture of wild edible plants, traditional hunting, and fishing [10, 11]. The geographical environment is critical in shaping the genetic structure of the population in forming landraces [12]. The formation of genetic landscapes of *Juglans regia* and *J. sigillata* in the Himalayan region is influenced by the geographical environment [13]. The formation of barley, wheat, and yak varieties in the Tibetan region is influenced by the plateau climate of the area [14, 15].

The breeding and domestication of landraces aim to adapt to the local environment and meet specific cultural needs, including plants, animals, and microorganisms [16, 17]. It differs from general cultivars or standardized breeds in that it exhibits distinct regional characteristics and interacts with local cultures [18, 19]. For example, in South Kalimantan, the cultivation of Bayar rice in tidal swamp rice fields [20]. Bovine yak is a unique breed adapted to the high-altitude environment that Tibetan people have domesticated in the Qinghai-Tibet Plateau region [21]. The diversity of landraces also promotes the biodiversity of landscape and cultural diversity. There is a significant connection between the conservation of local variety diversity and cultural preservation. The loss of specific variety rituals or formulations serves as an early warning sign of variety loss; likewise, the loss of local varieties can also lead to the loss of traditional practices. For example, as traditional knowledge among the Yi people in Liangshan, China gradually diminishes, their staple crop, buckwheat, as a local variety, is also disappearing concurrently. The loss of this local variety signifies a reduction in agricultural heritage and the gradual erosion of traditional Yi culture [22]. Despite conservation efforts, local crop varieties confront grave peril globally [23–25].

The preservation of local varieties primarily entails on-site and off-site conservation. Off-site conservation, synonymous with ex situ conservation, which has been shown to diminish the genetic diversity of farmer rice varieties, potentially compromising their adaptability to new ecological niches [26]. Moreover, gene bank-preserved seeds lack comprehensiveness. An evaluation of 25 major crops, including rice, barley, legumes, and fruits, collected for inclusion in gene banks revealed significant disparities among them, with rice at 60%, indicating that 40% of rice landraces remain unrepresented in gene banks [27]. In contrast, on-site conservation represents a dynamic safeguarding approach. This method exposes crops to the agricultural milieu, where they encounter threats such as pests and environmental stresses. While posing risks, on-site conservation fosters crop evolution and effectively sustains the genetic diversity of desirable agronomic traits in crops [28].

Farmers conserve landraces by continuing to cultivate and manage multiple crop populations within agricultural ecosystems, representing a process of co-selection by humans and nature [29]. This approach preserves the natural mutation and evolutionary diversity of crop resources amidst environmental changes and encompasses human selection and management. Ethnic customs and traditional practices play a pivotal role in safeguarding crop landraces and enhancing genetic diversit [30, 31]. As a staple food crop with a lengthy cultivation history, on-farm in situ conservation has yielded many rice landraces. By the conclusion of 2022, the National Crop Germplasm Bank had conserved over 100,000 rice landraces, with two-thirds comprising landraces (provided by the National Crop Germplasm Bank). The southwestern regions of China, including Guizhou, Yunnan, and Guangxi, represent the world's largest centers for rice genetic diversity and high-quality germplasm. A comparison of diversity between the same varieties stored in the National Gene Bank in 1980 and those collected from farmers in 2014, utilizing SSR analysis, revealed that farmer varieties exhibit more allele genes and higher genetic diversity. This suggests that on-farm conservation effectively fosters allelic variation and genetic diversity in rice landraces [32]. Kam Sweet Rice (KSR) is a unique rice variety bred and domesticated by the Dong ethnic group in China through their agricultural practices and cultural customs. Despite the continual replacement of rice landraces by new breeds, KSR remains the predominant cultivated variety among the Dong people due to its integral role in traditional diets, festival culture, and religious practices, ensuring its ongoing preservation and cultivation [33]. The abundant genetic diversity of farmer varieties in Xishuangbanna is also influenced by local cultural factors [34]. For instance, research by Xu et al. found that factors such as the inability of new varieties to adapt to local habitats and the preservation of traditional practices are critical reasons for the diversity conservation of local rice, wheat, and corn varieties upheld by ethnic minority farmers [35]. However, there is currently no systematic research on the conservation factors of rice landraces in Xishuangbanna and their impact on the inheritance of ethnic cultures.

Rice (*Oryza sativa* L.) serves as a principal staple food, furnishing over 20% of the caloric intake for more than half of the world’s population [36]. Southern China emerges as one of the primary hub of rice domestication. Yunnan, nestled in the southwest of China, emerges as the largest repository of genetic diversity of rice landraces and a bastion of superb genetic resources [37]. The Xishuangbanna Dai Autonomous Prefecture, nestled in the southwestern of Yunnan, serves as the epicenter for rice resource diversity and ethnic multiplicity in Yunnan, with diverse ethnic groups fostering a spectrum of rice resources, constituting approximately 20% of the province’s total resources. The region, characterized by elevated temperatures and copious rainfall, hosts the Dai people alongside over ten other indigenous ethnic groups, such as Bulang, Yao, and Hani. Favorable climatic conditions, in tandem with ethnic cultures, underpin the genetic diversity, uniqueness, and irreplaceability of landraces. However, the existing array of landraces in the Xishuangbanna region and the factors influencing their loss and conservation remain unclear.

Glutinous rice, a sticky variant of cultivated rice, owes its characteristic to a high amylopectin content in the embryo starch (≥ 98%), presenting a milky white appearance in the endosperm [38]. Culturally significant across Asia and serving as a staple in East Asian diets, glutinous rice emerges as a predominant variety in Xishuangbanna. The Dai people, constituting 35% of the region’s populace and serving as the primary ethnic group in Xishuangbanna, have played an indispensable role in preserving glutinous rice varieties. The Dai people in Xishuangbanna mainly inhabit river valleys and relatively flat terrain, where the water and temperature conditions are highly suitable for rice cultivation. From Sangmudi to the late 1990s, glutinous rice remained the main type of rice grain. Nonetheless, entering the twenty-first century, the tradition of glutinous rice as a staple food among the Dai has seen an irreversible decline due to multifaceted political, economic, and cultural influences [39]. During the Yunnan rice germplasm survey from 1978 to 1981, 123 glutinous rice varieties were cultivated locally, yet by 2007, only 22 varieties persisted [40]. However, despite this decline, the consumption of glutinous rice as a primary food source persists in some Dai villages, particularly among the elderly in their daily diets. Glutinous rice holds symbolic significance during significant festivals, life ceremonies, weddings, funerals, and housewarming events, serving as gifts and offerings that symbolize connections between individuals, communities, and the divine. Glutinous rice not only sustains material life but also embodies cultural symbolism within Dai communities [41]. Evidently, this trend undermines the preservation and safeguarding of rice varieties, diminishing the groundwork for glutinous rice breeding and rendering biodiversity conservation exceedingly fragile.

In this study, we selected 19 ethnic villages across one city and two counties in Xishuangbanna, collecting rice landraces preserved through on-farm conservation by local farmers. We recorded and compared the agronomic traits of different rice seeds, elucidating the diversity of rice landraces and their correlation with geographic location and ethnicity in the region. Focusing on glutinous rice as the primary research subject, we illuminated the present status of glutinous rice resource protection within the dietary, festival, religious sacrificial, and agricultural cultures of the Dai people. This study holds significant importance in better protecting and utilizing rice landraces in Xishuangbanna, advancing on-farm conservation efforts, thereby playing a crucial role in preserving ethnic cultures and customs.

## Methods

### Study site

Xishuangbanna, situated in the southwest of Yunnan Province, stands as one of the eight autonomous prefectures in Yunnan, comprising two counties and one city. Geographically, it occupies the northern periphery of the tropics, located south of the Tropic of Cancer (between 21° 10′ to 22° 40′ N, and 99° 55′ to 101° 50′ E), sharing borders with Puer City to the northeast, Laos to the southeast, and Myanmar to the southwest [42]. Xishuangbanna experiences a warm and humid climate year-round, lacking distinct seasons but instead characterized by a dry and wet season. The average annual temperature in Xishuangbanna hovers around 21 °C, with annual rainfall ranging from 1136 to 1513 mm. Elevations vary from 477 to 2429 m. With ample water resources and efficient irrigation, the region boasts geographical and climatic conditions conducive to rice cultivation [43].

As of 2022, Xishuangbanna’s permanent resident population stands at 1.308 million people, hosting 13 ethnic groups, including the Dai, Han, Hani, Yi, Lahu, Bulang, Jinuo, Yao, Miao, Hui, Wa, Zhuang, and Jingpo. The Dai people comprised 334,400 individuals, constituting 32.8% of the registered population.

Xishuangbanna cultivate various crops, including rice, corn, soybeans, and potatoes, among others, with rice cultivation spanning 30% of the total cultivated area. Glutinous rice cultivation in Xishuangbanna boasts a lengthy history, which is particularly cherished by the Dai people. In 1964, the Dai community in Xishuangbanna cultivated glutinous rice in over 90% of all rice planting areas; however, the cultivation area has markedly declined over the past three decades. Statistics indicate that in 2014, the total rice planting area reached 9779 ha, with glutinous rice accounting for merely 286 ha, representing 3% of the total planting area [41].

### Sample collection

**Fig. 1:**
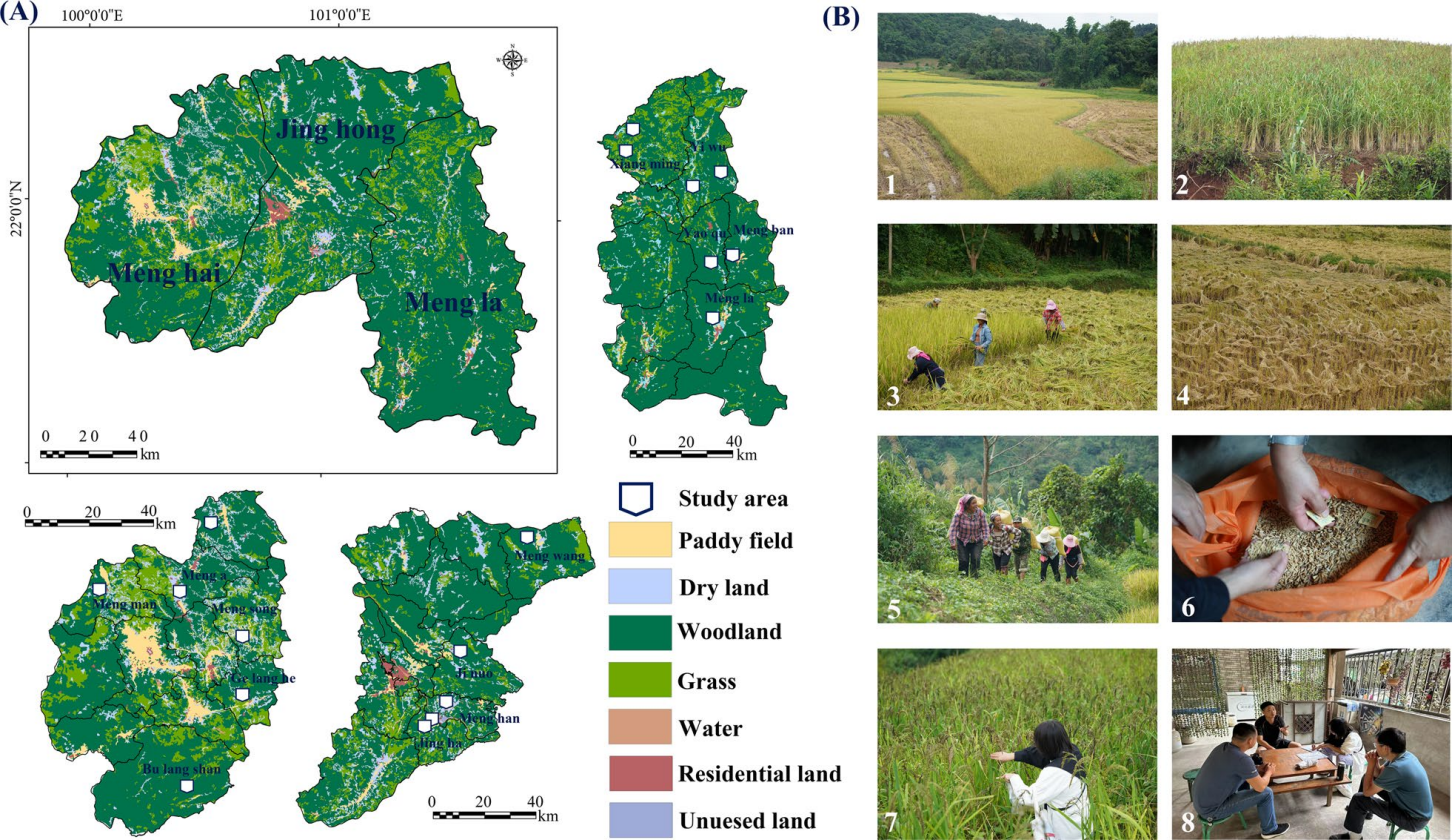
Distribution of study locations (**A**) and field investigation images (**B**). B1: lowland rice; B2: upland rice; B3: local people harvest rice; B4: drying rice in the field; B5: the dried rice is carried out of the field B6: rice after threshing; B7: field sampling; B8: farm interview

Adhering to the principles of selecting ethnobotanical survey points based on ecological environment, cultural preservation completeness, external influence, and other pertinent factors [44], an ethnobotanical survey was undertaken from October to November 2023 across 18 townships spanning Menghai, Mengla, and Jinghong (Fig. 1). Prior to commencing the survey and collection activities, coordination took place with the Xishuangbanna Agricultural Science Research Institute to pinpoint villages with extensive cultivation of local varieties, designating these areas as potential survey sites. A total of 70 rice landraces were gathered during the survey. The identification process commenced with farmers recognizing the varieties, which were subsequently validated by rice resource experts (Han Longzhi) at the National Crop Germplasm Bank before being preserved. The names of the gathered rice landraces and fundamental information regarding the survey locations, are detailed in Table 1.

**Table 1:** Basic information and characteristics of landraces collected

Sample number	Names	County	Township	Village	Longitude	Latitude	Altitude(m)	Nation	Indica/ Japonica	Glutinous/ Non-glutinous	Lowland/ Upland	Grain shape	Hull color	Hemp shell	Awn	Brown rice color	1000-grain weight (g)	Grain length (mm)	Grain width (mm)	Length-width ratio
1	Kongmingshangu	Mengla	Xiangming	Anle	101.19	22.09	1398	Yi	Indica	Non-glutinous	Lowland	Oval shape	Yellow like stem	No	No	White	23.96	8.00	2.58	3.12
2	Laowogu1	Mengla	Xiangming	Anle	101.19	22.09	1398	Yi	Japonica	Non-glutinous	Lowland	Oval shape	Brown	Yes	No	White	22.03	7.09	2.74	2.60
3	Xishagu	Mengla	Xiangming	Qiaotou	101.23	22.18	1293	Dai	Japonica	Glutinous	Lowland	Oval shape	Yellow	Yes	No	White	22.81	6.75	2.89	2.34
4	Dianlong201	Mengla	Xiangming	Qiaotou	101.23	22.18	1293	Dai	Indica	Glutinous	Lowland	Oval shape	Yellow like stem	No	No	White	24.52	7.34	2.95	2.50
5	Shoupeng(ying)	Mengla	Yiwu	Manla	101.47	21.98	1274	Dai	Indica	Glutinous	Lowland	Oval shape	Yellow like stem	No	No	White	25.86	8.37	2.58	3.26
6	Shoupeng(ruan)	Mengla	Yiwu	Manla	101.47	21.98	1274	Dai	Indica	Glutinous	Lowland	Oval shape	Yellow like stem	No	No	White	25.57	8.58	2.62	3.28
7	Heikenuo	Mengla	Yiwu	Mahei	101.60	22.03	1187	Yao	Japonica	Glutinous	Upland	Oval shape	Purple-black	No	Yes	White	32.91	9.40	3.55	2.68
8	Shangmengu	Mengla	Yiwu	Mahei	101.60	22.03	1187	Yao	Japonica	Non-glutinous	Upland	Oval shape	Reddish brown	Yes	No	White	31.09	8.58	3.38	2.56
9	Zinuo	Mengla	Yiwu	Mahei	101.60	22.03	1187	Yao	Indica	Glutinous	Upland	Oval shape	Purple	No	Yes	Purple	18.63	8.85	3.13	2.84
10	Mahei1	Mengla	Yiwu	Mahei	101.60	22.03	1187	Yao	Japonica	Glutinous	Upland	Oval shape	Reddish brown	No	No	White	25.10	9.48	3.58	2.66
11	Mahei2	Mengla	Yiwu	Mahei	101.60	22.03	1187	Yao	Japonica	Glutinous	Upland	Oval shape	Yellow like stem	No	No	White	26.29	8.02	3.25	2.48
12	Maxiangu	Mengla	Yiwu	Mahei	101.60	22.03	1187	Yao	Japonica	Non-glutinous	Upland	Oval shape	Reddish brown	Yes	No	White	31.29	8.76	3.37	2.61
13	Mahei3	Mengla	Yiwu	Mahei	101.60	22.03	1187	Yao	Japonica	Non-glutinous	Upland	Oval shape	Reddish brown	Yes	No	White	29.11	8.43	3.19	2.65
14	Jizigu	Mengla	Yiwu	Mahei	101.60	22.03	1187	Yao	Japonica	Non-glutinous	Upland	Oval shape	Yellow	No	No	White	27.49	8.11	3.26	2.50
15	Laowogu2	Mengla	Yaoqu	Sharen	101.54	21.72	779	Yao	Indica	Glutinous	Lowland	Thin long shape	Yellow like stem	No	No	White	32.72	9.28	3.01	3.11
16	Sharen1	Mengla	Yaoqu	Sharen	101.54	21.72	779	Yao	Indica	Non-glutinous	Lowland	Thin long shape	Yellow like stem	No	No	White	26.81	9.17	2.38	3.87
17	Sharen2	Mengla	Yaoqu	Sharen	101.54	21.72	779	Yao	Indica	Non-glutinous	Lowland	Thin long shape	Yellow like stem	No	No	White	26.13	8.85	2.49	3.59
18	Sharen3	Mengla	Yaoqu	Sharen	101.54	21.72	779	Yao	Indica	Non-glutinous	Lowland	Thin long shape	Yellow like stem	No	No	White	26.84	8.81	2.52	3.52
19	Sharen4	Mengla	Yaoqu	Sharen	101.54	21.72	779	Yao	Indica	Glutinous	Lowland	Oval shape	Yellow like stem	No	No	White	35.87	9.58	3.03	3.19
20	Sharen5	Mengla	Yaoqu	Sharen	101.54	21.72	779	Yao	Indica	Non-glutinous	Lowland	Thin long shape	Yellow like stem	No	No	White	25.50	8.44	2.57	3.30
21	Gaogu	Mengla	Yaoqu	Sharen	101.54	21.72	779	Yao	Indica	Non-glutinous	Lowland	Oval shape	Yellow like stem	No	No	White	24.72	8.14	2.55	3.21
22	Shangmengu2	Mengla	Yaoqu	Sharen	101.54	21.72	779	Yao	Japonica	Non-glutinous	Lowland	Oval shape	Reddish brown	No	No	White	31.03	8.50	3.25	2.64
23	Miaojiugu	Mengla	Yaoqu	Sharen	101.54	21.72	779	Yao	Indica	Non-glutinous	Lowland	Oval shape	Yellow like stem	No	No	White	29.73	8.35	2.87	2.93
24	Xiongdigu	Mengla	Yaoqu	Sharen	101.54	21.72	779	Yao	Indica	Non-glutinous	Lowland	Oval shape	Yellow like stem	No	No	White	25.20	7.33	2.90	2.54
25	Mabagu	Mengla	Yaoqu	Sharen	101.54	21.72	779	Yao	Indica	Non-glutinous	Lowland	Thin long shape	Yellow like stem	No	No	White	26.20	8.65	2.64	3.36
26	Dianbanhong	Mengla	Yaoqu	Sharen	101.54	21.72	779	Yao	Indica	Non-glutinous	Lowland	Oval shape	Yellow	No	No	White	27.76	8.24	2.89	2.87
27	Gaogu	Mengla	Yiwu	Mannai	101.47	21.98	2439	Dai	Indica	Glutinous	Lowland	Oval shape	Yellow like stem	No	No	White	25.95	8.23	2.69	3.08
28	Gaoguzi	Mengla	Mengban	Mengban	101.64	21.73	2453	Dai	Japonica	Non-glutinous	Lowland	Oval shape	Yellow like stem	No	No	White	26.56	7.86	3.01	2.64
29	Zinuo	Mengla	Mengban	Mengban	101.64	21.73	2453	Dai	Japonica	Glutinous	Lowland	Oval shape	Brown	Yes	No	Purple	24.47	8.58	3.29	2.62
30	Baihaogu	Mengla	Mengban	Mengban	101.64	21.73	2453	Dai	Japonica	Non-glutinous	Lowland	Oval shape	Yellow like stem	No	No	White	31.55	8.22	3.51	2.37
31	Nanbanghonggu	Mengla	Mengla	Mandan	101.57	21.49	660	Dai	Japonica	Non-glutinous	Lowland	Oval shape	Yellow	Yes	No	White	20.00	8.23	3.52	2.37
32	Nanbanggu	Mengla	Mengla	Mandan	101.57	21.49	660	Dai	Indica	Non-glutinous	Lowland	Thin long shape	Yellow like stem	No	No	White	29.57	8.91	2.59	3.46
33	Nanbangshangu	Mengla	Mengla	Mandan	101.57	21.49	660	Dai	Japonica	Non-glutinous	Lowland	Oval shape	Yellow	Yes	No	White	27.17	8.29	3.01	2.77
34	Haonuo	Jinghong	Menghan	Manyuan	100.96	21.91	2473	Dai	Indica	Glutinous	Lowland	Oval shape	Yellow like stem	No	No	White	27.93	9.02	2.92	3.11
35	Haoanmanle	Jinghong	Menghan	Manyuan	100.96	21.91	2473	Dai	Indica	Glutinous	Lowland	Thin long shape	Yellow like stem	No	No	White	31.76	9.36	2.86	3.31
36	Xihonggu	Menghai	Bulangshan	Banzhang	100.94	21.85	2480	Bulang	Japonica	Non-glutinous	Upland	Oval shape	Yellow like stem	No	No	Red	18.29	6.64	2.62	2.56
37	Zinuo	Menghai	Bulangshan	Banzhang	100.94	21.85	2480	Bulang	Japonica	Glutinous	Upland	Oval shape	Purple	No	No	Purple	24.09	7.90	3.28	2.42
38	Xihonggu	Jinghong	Jinuoshan	Baya	100.99	22.06	2458	Jinuo	Japonica	Glutinous	Lowland	Oval shape	Yellow like stem	No	No	Red	18.32	6.69	2.67	2.52
39	Hongliandaogu	Jinghong	Jinuoshan	Baya	100.99	22.06	2458	Jinuo	Japonica	Glutinous	Lowland	Oval shape	Yellow like stem	No	No	Red	23.17	7.52	2.75	2.76
40	Zinuogu	Jinghong	Jinuoshan	Baya	100.99	22.06	2458	Jinuo	Japonica	Glutinous	Lowland	Oval shape	Yellow like stem	No	No	Purple	25.45	7.95	3.29	2.43
41	Ximenggu	Jinghong	Jinuoshan	Baya	100.99	22.06	2458	Jinuo	Japonica	Non-glutinous	Lowland	Oval shape	Yellow like stem	No	No	White	19.76	6.70	2.76	2.45
42	Manhonggu	Jinghong	Mengwang	Mengwang	101.29	22.49	2401	Dai	Indica	Non-glutinous	Lowland	Thin long shape	Yellow	No	No	White	22.75	8.16	2.23	3.69
43	Baishangu	Jinghong	Jingha	Geniu	100.93	21.84	2482	Hani	Japonica	Non-glutinous	Lowland	Oval shape	Yellow	Yes	No	White	26.63	7.57	3.29	2.32
44	Dahonggu	Jinghong	Jingha	Geniu	100.93	21.84	2482	Hani	Japonica	Glutinous	Lowland	Oval shape	Yellow	No	No	Red	31.97	7.59	3.28	2.33
45	Honggu1	Jinghong	Jingha	Geniu	100.93	21.84	2482	Hani	Japonica	Non-glutinous	Lowland	Oval shape	Yellow like stem	No	No	Red	18.24	6.88	2.74	2.52
46	Xiaohonggu1	Jinghong	Jingha	Geniu	100.93	21.84	2482	Hani	Japonica	Non-glutinous	Lowland	Oval shape	Yellow like stem	No	No	Red	26.49	7.75	2.94	2.66
47	Xiaohonggu2	Menghai	Gelanghe	Henanshangzhai	100.57	21.86	2500	Hani	Japonica	Non-glutinous	Lowland	Short round shape	Purple-black	No	Yes	Red	21.57	6.59	3.34	1.98
48	Xiaohonggu3	Menghai	Gelanghe	Henanshangzhai	100.57	21.86	2500	Hani	Japonica	Non-glutinous	Lowland	Short round shape	Yellow like stem	No	No	Red	20.36	6.47	3.10	2.12
49	Honggu2	Menghai	Gelanghe	Henanshangzhai	100.57	21.86	2500	Hani	Indica	Non-glutinous	Lowland	Oval shape	Yellow like stem	No	No	Red	29.00	7.77	2.88	2.72
50	Manfang1	Menghai	Mengsong	Manfang	100.56	22.05	2482	Dai	Indica	Glutinous	Lowland	Oval shape	Yellow	No	No	White	29.72	8.48	3.13	2.72
51	Hongxiangnuo	Menghai	Mengsong	Manfang	100.56	22.05	2482	Dai	Indica	Glutinous	Upland	Oval shape	Brown	Yes	No	Red	24.17	7.90	2.69	2.97
52	Manfang 2	Menghai	Mengsong	Manfang	100.56	22.05	2482	Dai	Indica	Non-glutinous	Upland	Oval shape	Yellow like stem	No	No	Red	25.56	7.66	2.88	2.68
53	Luojiagu	Menghai	Mengsong	Manfang	100.56	22.05	2482	Dai	Japonica	Non-glutinous	Lowland	Oval shape	Yellow like stem	No	No	White	24.00	6.92	2.74	2.55
54	Huipagu	Menghai	Mengsong	Manfang	100.56	22.05	2482	Dai	Japonica	Non-glutinous	Lowland	Oval shape	Yellow like stem	No	No	White	20.39	6.95	2.73	2.56
55	Dibaigu	Menghai	Mengsong	Manfang	100.56	22.05	2482	Dai	Japonica	Non-glutinous	Lowland	Oval shape	Yellow	No	No	White	31.68	7.95	3.59	2.22
56	Xiaohonggu	Menghai	Mengsong	Manfang	100.56	22.05	2482	Dai	Japonica	Non-glutinous	Lowland	Short round shape	Brown	No	Yes	Red	22.67	6.17	3.06	2.03
57	Gan3	Menghai	Mengsong	Manfang	100.56	22.05	2482	Dai	Indica	Glutinous	Lowland	Oval shape	Yellow	No	No	White	28.24	8.50	3.12	2.75
58	Daxianggu	Menghai	Mengman	Bandao	100.12	22.17	2496	Lahu	Japonica	Non-glutinous	Lowland	Oval shape	Brown	Yes	No	White	29.36	7.76	3.49	2.24
59	Bandao1	Menghai	Mengman	Bandao	100.12	22.17	2496	Lahu	Japonica	Non-glutinous	Lowland	Short round shape	Purple-black	Yes	Yes	White	22.50	6.84	3.16	2.18
60	Miandiangu	Menghai	Mengman	Bandao	100.12	22.17	2496	Lahu	Japonica	Non-glutinous	Lowland	Oval shape	Yellow	No	No	White	31.60	7.92	3.57	2.23
61	ming su qie	Menghai	Mengman	Bandao	100.12	22.17	2496	Lahu	Japonica	Non-glutinous	Lowland	Ovum shape	Yellow like stem	No	No	White	24.44	6.84	3.14	2.20
62	Puergu谷	Menghai	Mengman	Bandao	100.12	22.17	2496	Lahu	Japonica	Non-glutinous	Lowland	Oval shape	Yellow	No	No	White	29.24	8.04	3.24	2.49
63	Bandao2	Menghai	Mengman	Bandao	100.12	22.17	2496	Lahu	Japonica	Non-glutinous	Lowland	Oval shape	Brown	Yes	No	White	30.53	7.68	3.37	2.30
64	Duodaguo	Menghai	Bulangshan	Akexinzhai	100.41	21.58	1234	Bulang	Japonica	Non-glutinous	Lowland	Oval shape	Yellow like stem	No	No	White	22.32	6.99	3.04	2.32
65	Handihongnuomi	Menghai	Bulangshan	Akexinzhai	100.41	21.58	1234	Bulang	Japonica	Glutinous	Lowland	Oval shape	Reddish brown	No	No	White	28.98	8.21	3.32	2.49
66	Taidihongmi	Menghai	Bulangshan	Akexinzhai	100.41	21.58	1234	Bulang	Japonica	Non-glutinous	Lowland	Oval shape	Yellow like stem	No	No	Red	21.23	6.63	2.91	2.31
67	Kaonuozhang	Menghai	Bulangshan	Akexinzhai	100.41	21.58	1234	Bulang	Japonica	Glutinous	Lowland	Oval shape	Yellow like stem	No	No	White	24.64	7.20	3.17	2.29
68	HongmiRed米	Menghai	Bulangshan	Akexinzhai	100.41	21.58	1234	Bulang	Japonica	Non-glutinous	Lowland	Oval shape	Yellow like stem	No	No	Red	23.13	6.68	2.83	2.40
69	Xiaohonggu4	Menghai	Mengwang	Basan	100.47	22.38	796	Dai	Indica	Glutinous	Lowland	Oval shape	Yellow like stem	No	No	Red	31.13	7.97	3.00	2.69
70	Dihonggu	Menghai	Menga	Chengzi	100.33	22.18	2483	Lahu	Japonica	Non-glutinous	Lowland	Oval shape	Yellow like stem	No	No	Red	25.32	6.91	3.15	2.22

### Field survey and data collection

Interviews were conducted utilizing free listing, semi-structured interviews, key informant interviews, and participatory observation [45]. The selected interviewees represented various ethnic groups, including Dai, Yao, Bulang, Hani, Yi, and Jinuo. During the survey, interviews were conducted with 120 respondents using the snowball sampling method. Among them, there were 67 males and 53 females, ranging in age from 18 to 76. Occupations included farming, salaried temporary work, trading, and student, with educational backgrounds including illiterate, primary school, middle school, high school or above. We first contacted personnel from the local agricultural research institute. Then, they contacted the village chiefs who still possess rice landraces in the Xishuangbanna region. Upon arrival at the collection site, we first interviewed the village chiefs. Subsequently, the village chiefs recommended other individuals from their villages who were knowledgeable about rice landraces and ethnic cultures. We then conducted interviews with them.

There were 27 key informants identified for this study. According to the purpose of this research, key informants included the director of the local agricultural research institute who has been engaged in agricultural management for nearly 20 years, village chiefs from various villages, indigenous people familiar with local ethnic cultures, and farmers with extensive experience in rice cultivation and management. Before the interviews, participants were informed of the project's purpose and their consent was obtained. Survey locations included farmers' homes, fields, and streets.

Each interviewee underwent a semi-structured interview covering the following topics: informant's information (gender, ethnicity, age, education, occupation), characteristics of landraces (local name, agronomic features, seed sources, altitude of rice cultivation, awn color and length, hull and grain color), utilization of landraces (utilization of rice in traditional practices including dietary, medicinal, agricultural, festival, and sacrificial), and the current status of landraces preservation (loss of landraces, reasons for loss, and local conservation policies). Detailed questions for each topic are listed in Table 2.

**Table 2:** Questions in semi-structured interviews

	Questions	Answers	
Demographic characteristic of respondents	Q1: Gender	A: Male	B: Female
	Q2: Ethnicity		
	Q3: Age		
	Q4: Education	A: Illiterate	B: Primary school
		C: Middle school	D: High school or above
	Q5: Main occupation	A: Farming	B: Salaried temporary work
		C: Trading	D: Student
Characteristics of landraces	Q1: How many types of landraces do you have in your household? Q2: For each type of landraces, inquire about the following agronomic characteristics, including name, lowland/upland rice, planting altitude, grain shape, size, length, width, awn color, hull color, grain color, and seed sources		
Use of landraces	Q1: What are the reasons for choosing to cultivate these landraces?	A: Outstanding characteristics	B: Economic value
		C: Cultural value	D: Daily consumption
	Q2: Apart from daily consumption, are these landraces also used in other traditional festivals?	A: Yes	B: No
	Q3: Which traditional activities or festivals do you use the landraces in, and what is the significance behind it?		
Conservation status of landraces	Q1: What is the current proportion of cultivation for landraces?		
	Q2: How much loss has occurred in the landraces compared to before?		
	Q3: What are the reasons for the loss of these landraces?		

Before and after the field survey, we also conducted literature research by reviewing publicly available journals, papers, books, local chronicles, and unpublished local statistical data. This helped us understand Xishuangbanna's natural geographical conditions and the landraces' distribution and changes. Additionally, these sources provided information about the Dai ethnic group's dietary habits, festival activities, and other customs. This knowledge deepened my understanding of the current status of landraces and traditional practices in Xishuangbanna and the protection of these two aspects.

### Agronomic trait statistics

Referring to the technical specifications outlined for rice germplasm resource identification by Han et al. in 2021 [46], the agronomic traits of seeds collected from the 70 landraces were observed, systematically recorded, and photographed [47]. The methods and standardization of recording these traits are elaborated in Table 2. A total of 12 traits were recorded, comprising eight first-class traits and four second-class traits. The first-class traits were qualitative and encompassed indica/japonica classification (T1), glutinous/non-glutinous distinction (T2), lowland/upland preference (T3), grain shape (T4), hull color (T5), presence of a hemp shell (T6), awn presence (T7), and brown rice color (T8). Conversely, the second-class traits were quantitative, including 1000-grain weight (T9), grain length (T10), grain width (T11), and length–width ratio (T12) (Table 3).

**Table 3:** Primary features, standards, and assignments of agronomic traits across landraces

Agronomic traits	Phenotype
Indica/japonica (T1)	Indica (1); Japonica (2)
Glutinous/non-glutinous (T2)	Non-glutinous (1); Glutinous (2)
Lowland/upland (T3)	Lowland (1); Upland (2)
Grain shape (T4)	Short round shape (1); Ovum shape (2); Oval shape (3); Thin long shape (4)
Hull color (T5)	Yellow like stem (1); Yellow (2); Brown (3); Reddish brown (4); Purple (5); Purple-black (6)
Hemp shell (T6)	Yes (1); No (2)
Awn (T7)	Yes (1); No (2)
Brown rice color (T8)	White (1); Red (2); Purple (3)
1000-grain weight (T9)	Weight of 1000 pure seeds in dry air (g)
Grain length (T10)	Ripe and full grain length (mm)
Grain width (T11)	Width of the widest part on either side of the inner and outer glume of the mature full grain (mm)
Length–width ratio (T12)	Ratio of grain length to width

Upon assigning values and standardizing the 12 agronomic traits, a rigorous statistical analysis was conducted using SPSS 20.0, encompassing mean ($\overline{X}$), standard deviation (*δ*), range, maximum value, minimum value, and coefficient of variation. Following Li et al.’s methodology, the quantitative traits of all materials were quantified based on the mean and standard deviation, categorized into ten levels. These levels span from Level 1 (< $\overline{X}$ − 2δ) to Level 10 (≥ $\overline{X}$ + 2*δ*), with an interval of 0.5*δ* between each level [44]. The number of resources for each feature distribution of the first-class traits and the number of resources for each level of the second-class traits were statistically analyzed. The Shannon–Wiener diversity index (*H*′) was calculated for each of the 12 traits, using the formula as follows:

$$H^{\prime}={\sum }_{i=1}^{S} {P}_{i}\mathrm{ ln}{P}_{i}$$

where ${P}_{i}$ represents the percentage of resources in the *i* level of a trait to the total number of resources, ln denotes a natural logarithm, and *S* is the total number of resources.

### Cluster analysis of landraces

The correlation among the 12 traits was computed utilizing the Pearson method within the psych package of R (v4.1.3) [48]. Subsequently, for pairs of traits demonstrating significant correlation (*p* < 0.05), one of the correlated traits was judiciously chosen for elimination. With the retained traits, a cluster analysis of the 70 landraces was systematically performed, leveraging the UPGMA method through the vegan package [49], and the plot was generated using the ggplot2 package [50].

## Results

### Diversity of landraces in Xishuangbanna

**Fig. 2:**
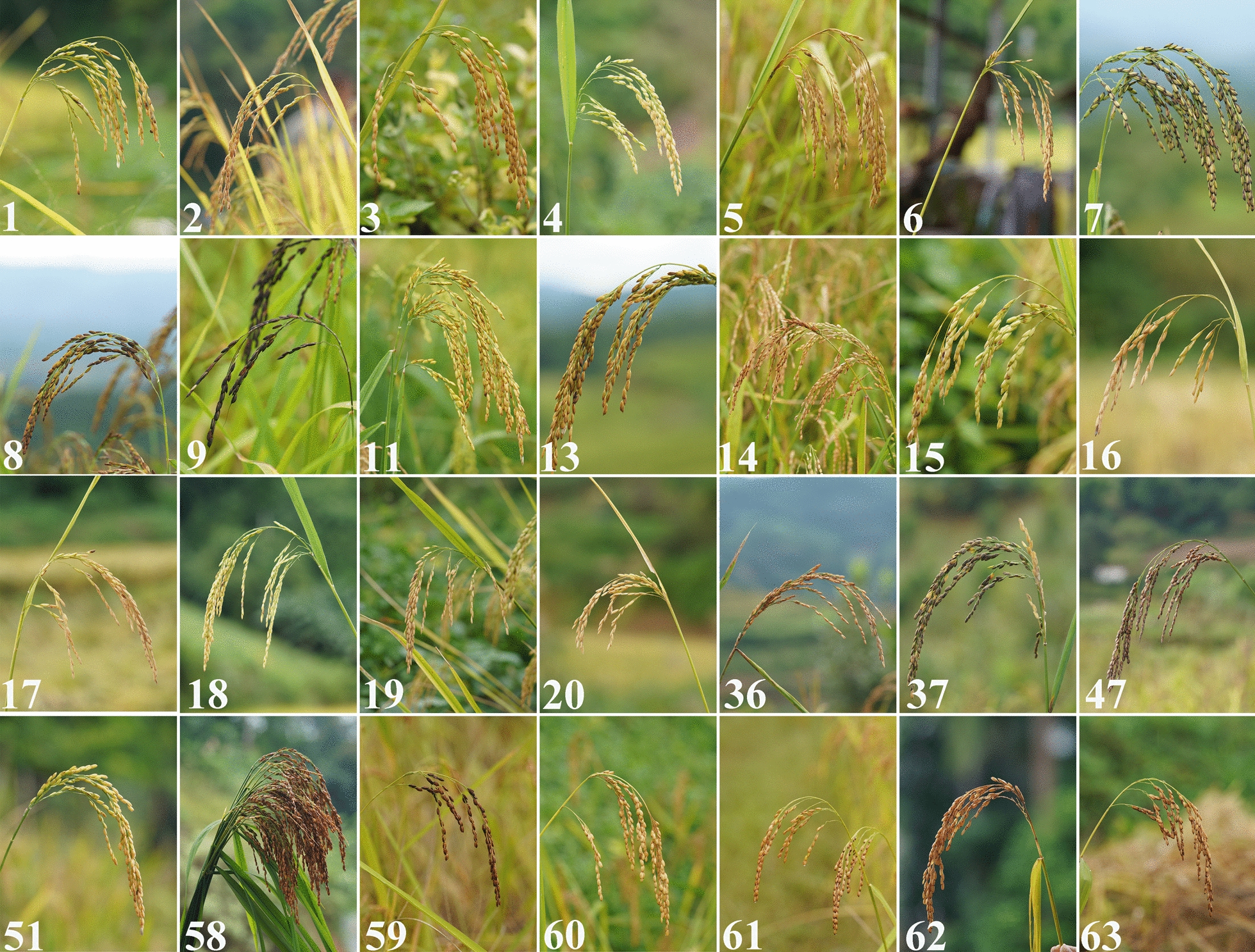
Display of rice landraces varieties in Xishuangbanna

**Fig. 3:**
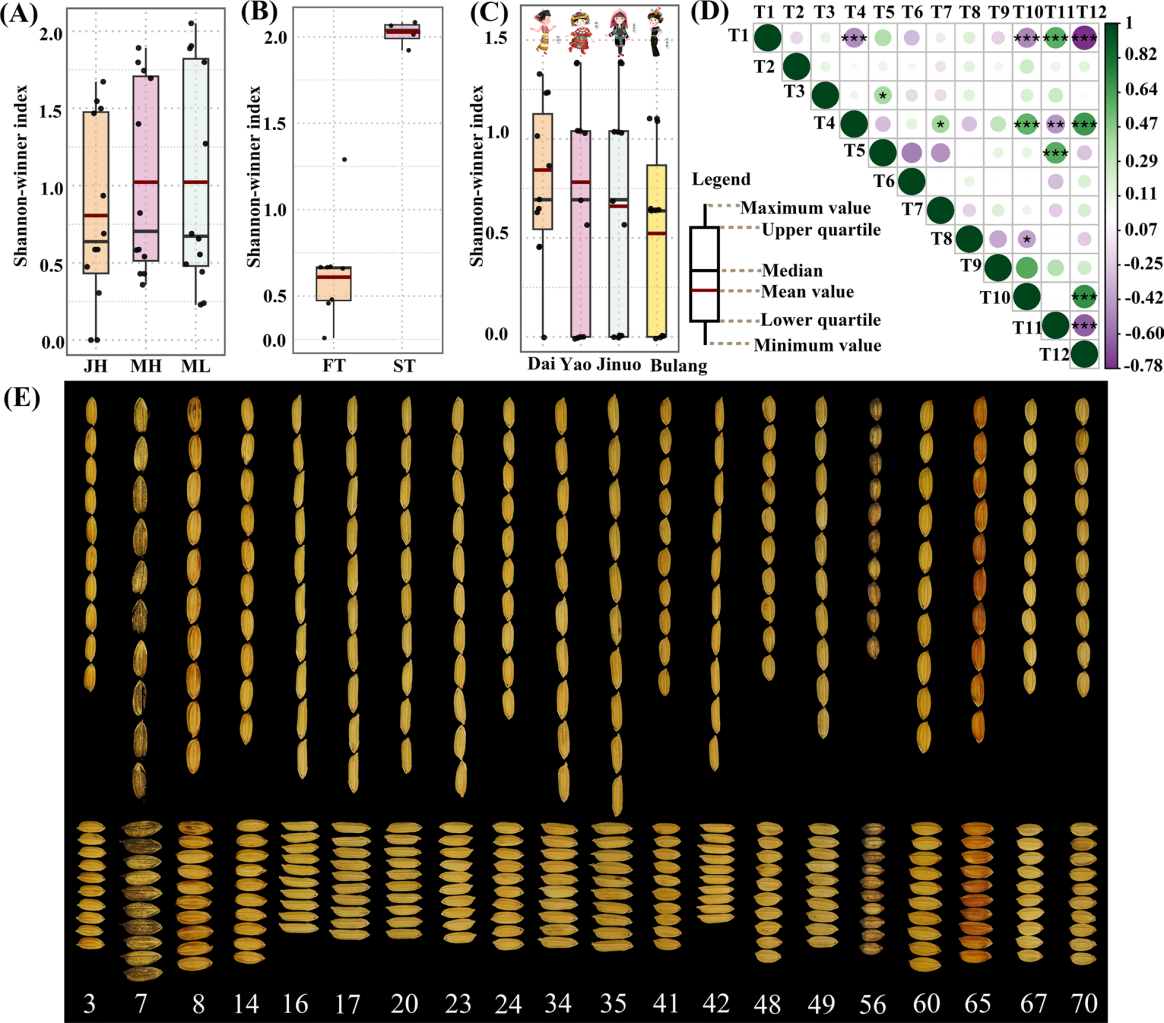
Diversity and Correlation of Phenotypic Traits. **A** Evaluation of regional *H*′, JH (Jinghong), MH (Menghai), ML (Mengla); **B** Assessment of H' between first-class traits (FT) and second-class traits (ST); **C** Comparison of H' in glutinous rice resources across different ethnic groups; **D** Pearson correlation analysis between traits, with circle size indicating *p*-values (***p < 0.001, **p < 0.01, *p < 0.05), and circle color representing the magnitude of the correlation coefficient; **E** Depiction of seed diversity for notable resources

A total of 70 landraces under the on-farm conservation were obtained from 20 villages across 18 townships of Menghai, Mengla, and Jinghong (Table 1). The field collection of rice landraces is depicted in Fig. 2. Diversity differences across regions, traits, and ethnicities were examined by calculating the Shannon–Wiener index, with results presented in Fig. 3.

1. Regional Diversity
  Within the two counties and one city, Mengla exhibited the highest tally of collected landraces, totaling 33, followed by Menghai with 26, and Jinghong with the least at 11. The mean Shannon–Wiener index of the 12 traits displayed consistent diversity in Menghai and Mengla at 1.02, while Jinghong exhibited lower diversity at 0.81 (Fig. 3A). This outcome suggests that the rice landraces in Jinghong tend to be more homogenous. Notably, two traits displayed Shannon–Wiener indices of 0, namely T3 and T7, signifying the absence of upland rice and all varieties lacking awns. Moreover, the hull color of rice varieties in Jinghong City showcased considerable uniformity, with only two types: stem yellow (8 samples) and yellow (3 samples).

(2) Trait Diversity
  Ethnic minorities in Xishuangbanna have cultivated diverse landraces well-suited to the local ecological environment, with disparities noted between varieties for both first-class traits (T1–T8) and second-class (T9–T12) (Fig. 3E). The Shannon–Wiener index results, depicted in Fig. 3B, underscore broader diversity among the second-class traits. The distribution of the 70 resources is detailed as follows: 27 indica, 43 japonica; 25 glutinous, 45 non-glutinous; 12 upland, 58 lowland; 13 hemp shell, 57 non-hemp shell; 5 with awns, 65 without awns. Among the first-class traits, grain shape, hull color, and brown rice color were relatively abundant. Hull color exhibited the highest diversity, encompassing yellow like stem (40 varieties), yellow (13 varieties), brown (6 varieties), reddish brown (6 varieties), purple (2 varieties), and purple-black (3 varieties). Notably, four distinct grain shapes were recorded, comprising a short round shape (4 varieties), an ovum shape (1 variety), an oval shape (56 varieties), and a thin long shape (9 varieties). In addition to 50 white varieties, 16 red varieties, and four purple varieties were collected. The distribution of the four purple varieties is specified as follows: from a Yao in Mengla (#9), from a Dai in Mengla (#29), from a Bulang in Menghai (#37), and from a Hani in Jinghong (#40).
  Statistical analysis of the information and number of resources across each level of the second-class traits unveiled significant differences among resources, albeit with a generally normal distribution. The range of 1000-grain weight spanned from 18.24 to 35.87 g, predominantly concentrated between 22 and 26 g, exhibiting the largest coefficient of variation (15.42%, Table 4). Only seven materials boasted grain lengths exceeding 9.00 mm, with the heaviest and longest resource being #19, collected in Mengla, featuring a 1000-grain weight of 35.87 g and a grain length of 9.58 mm. Conversely, the shortest grain length was attributed to #56 at 6.16 mm (Fig. 3E). Notably, #42 collected from a Dai family in Jinghong, exhibited a slender profile with the smallest grain width at 2.23 mm (Fig. 3E), while the widest grain width measured 3.58 mm (#55). The length–width ratio emerged as the second-class trait with the largest coefficient of variation, an essential consideration in breeding, with 16 materials boasting a ratio exceeding 3, among which only one was collected from a Yi family, seven from Dai families, and eight from Yao families.

**Table 4:** Statistical analysis results of second-class traits of rice landraces

Trait	Minimum value	Maximum value	Mean	SD	Coefficient of variation (%)
T9 (g)	18.24	35.87	26.11	3.99	15.42
T10 (mm)	6.17	9.58	7.91	0.83	10.61
T11 (mm)	2.23	3.58	3.01	0.32	10.78
T12	1.98	3.87	2.68	0.42	15.55

(3) Naming Diversity
  Grain shape, size, glutinousness, hull color, and brown rice color are significant criteria for local farmers’ folk classification and naming of landraces. For instance, #9, characterized by its glutinous nature and purple hull and brown rice color, earns the name “Zinuo” (Purple Glutinous). Similarly, #38, featuring thin grains and red-colored brown rice, is dubbed “Xihonggu” (Thin Red Grain), while #44, with larger grains and red-colored brown rice, is named “Dahonggu” (Big Red Grain). Apart from these characteristic-based names, different ethnic groups possess their naming customs. For instance, the Dai people often append the term “毫” (hao) to rice variety names, particularly in the case of glutinous rice. Moreover, aside from the agronomic traits surveyed in this study, the aroma post-cooking serves as another crucial criterion for local naming conventions. For example, #51, exhibiting a strong fragrance post-cooking and red coloration, earns the title “Hongxiangnuo” (Red Fragrant Glutinous).

(4) Diversity of Dai Ethnic Glutinous Rice
  The inventory of glutinous rice resources reveals cultivation among the Dai, Yao, Bulang, Jinuo, and Hani ethnic groups. Of the total samples collected, 25 (comprising 36%) were glutinous rice varieties, with Dai households contributing 12 samples, constituting 44%. The Yao ethnic group follows closely with six samples, while the Jinuo and Bulang ethnic groups each contribute three samples, and the Hani ethnic group offers only one sample. Assessing the diversity of glutinous rice among different ethnic groups using the Shannon–Wiener index (Fig. 3C), the results indicate that Dai ethnic group resources boast the highest average Shannon–Wiener index of 0.82, trailed by the Yao at 0.77, Jinuo at 0.64, and Bulang with the lowest at 0.53. This suggests that glutinous rice resources cultivated by Dai households exhibit higher diversity.

### Clustering of phenotypic traits

**Fig. 4:**
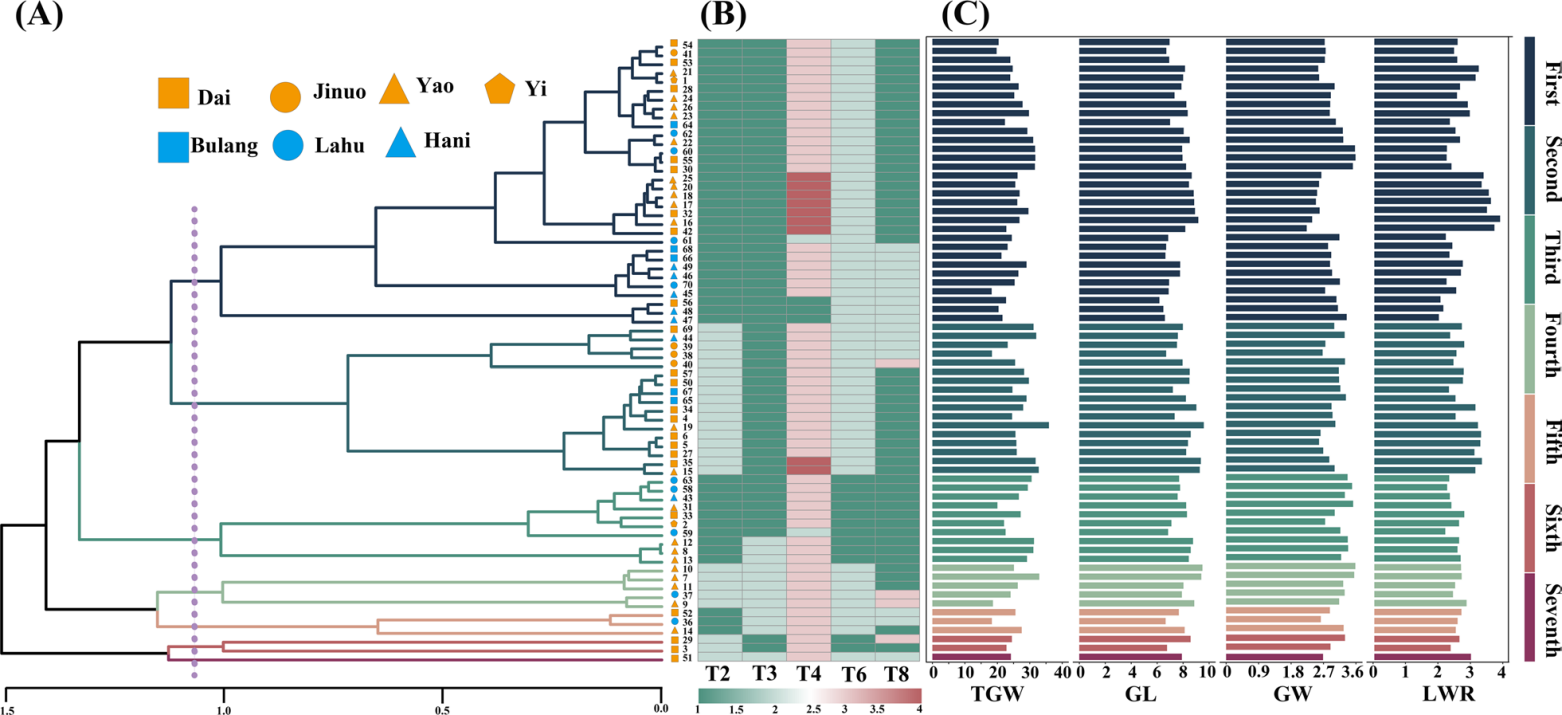
Phenotypic trait clustering diagram. **A** UPGMA method-based clustering results for T2, T3, T4, T6, T8, T9, T11; **B** Heatmap of the first-class traits used in cluster analysis; **C** the second-class traits, thousand grain weight (TGW), grain length (GL), grain width (GW), and length–width ratio (LWR)

To mitigate noise and address multicollinearity during the clustering process, a correlation analysis of agronomic traits was performed using the Pearson correlation coefficient, as depicted in Fig. 3D. For traits that were significantly correlated, only one trait was retained. The principle was to retain as many factors as possible, keeping both the first-class traits and the second-class traits. Eventually, T2, T3, T4, T6, T8, T9, and T11 were selected as input conditions for clustering. Based on genetic distance, the clustering analysis of agronomic traits of rice landraces indicated that at a genetic distance of 1.15, the 70 samples could be categorized into seven clusters (Fig. 4A). The heatmap of the first-class traits and the trait plot of the second-class traits reveal that the primary traits influencing clustering differentiation are the first-class traits.

The first-class seeds predominantly consisted of non-glutinous, lowland, and non-hemp shell rice. Conversely, the second-class seeds were predominantly glutinous, lowland, and non-hemp shell rices. The third class comprised non-glutinous, hemp shell rice, while the fourth class comprised glutinous upland rices. Additionally, the fifth class comprised non-glutinous upland rice, and the sixth class comprised glutinous hemp shell rices. The final class comprised only one material, a distinct rice resource attributed to the Dai people, characterized as glutinous, upland rice with an oval shape, hemp shell, and red-brown rice, further highlighting the uniqueness of Dai glutinous rice. Within each class, several subclasses were further divided based on other traits.

### The protection of glutinous rice in Dai traditional culture

**Fig. 5:**
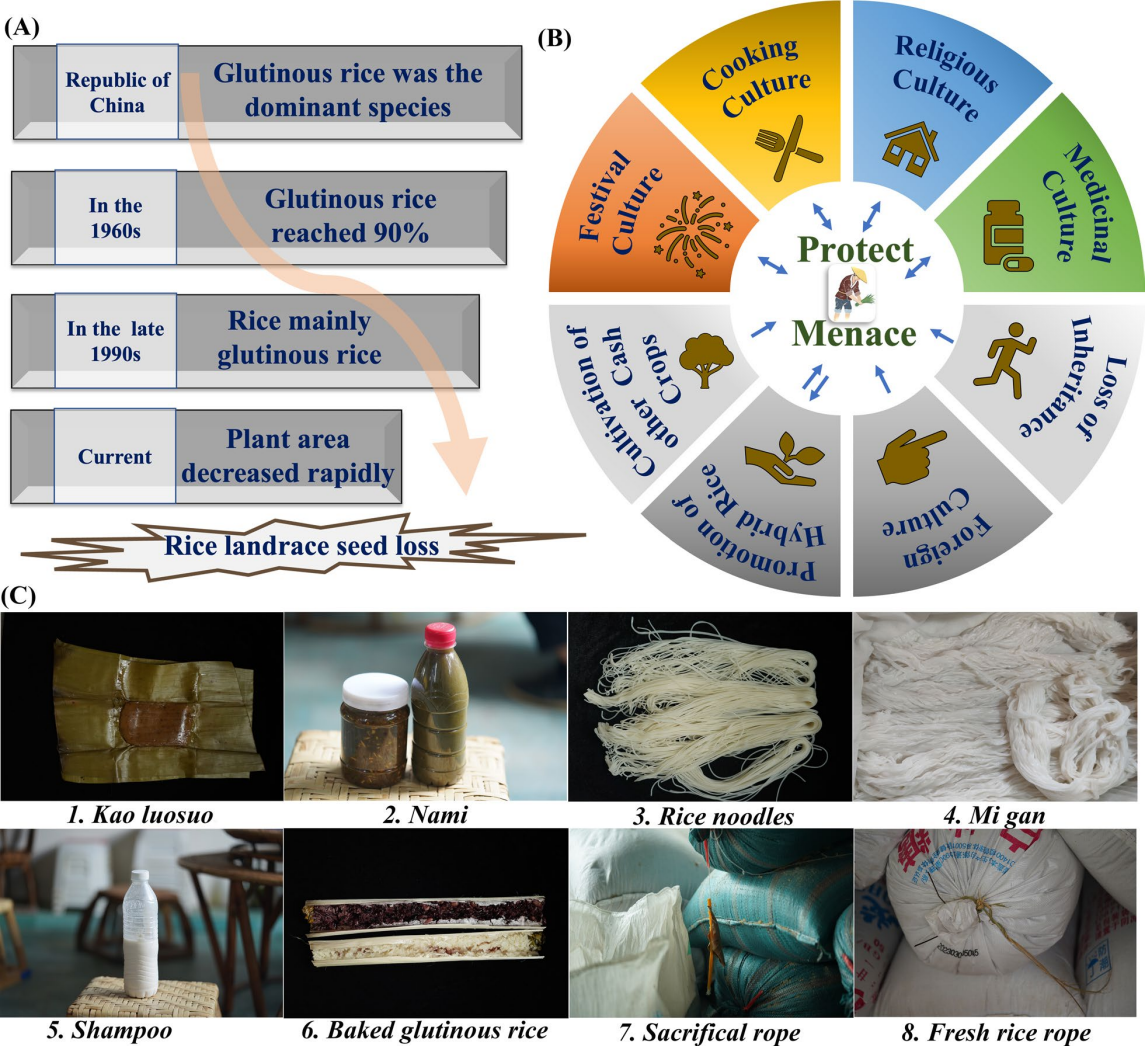
Relationship between glutinous rice in Xishuangbanna and Dai traditional culture **A** Alterations in the scale of glutinous rice cultivation in Xishuangbanna; **B** Protection and threats to landraces; **C** Dai glutinous rice and culture

The cultivation scale of glutinous rice in the Dai ethnic areas of Xishuangbanna has undergone four periods of change (Fig. 5A). However, the Dai people possess a wealthier resource of glutinous rice, and through our research, we have found that traditional culture plays a significant role in the protection of glutinous rice resources. Glutinous rice is indispensable in Dai dietary, medicinal, festival, and ritual cultures.

The initial cultivation of glutinous rice by the Dai people stemmed from its sticky texture, which made it convenient for consumption during labor-intensive activities. Today, many Dai villages prioritize glutinous rice cultivation to meet their dietary needs, with glutinous rice forming a staple part of their diet. Glutinous rice is not only consumed as a staple but is also used in various culinary preparations, such as Kao luosuo (Fig. 5C-1) and rice noodles (Fig. 5C-3), as well as in fermented foods and dips (Fig. 5C-2). This cultural reliance and fondness for glutinous rice have contributed to preserving glutinous rice varieties among the Dai population.

Beyond culinary uses, glutinous rice serves medicinal, ceremonial, and religious purposes within Dai culture. For instance, water from washing glutinous rice is used for hair care, and purple rice is valued for its medicinal properties, including its use to enrich blood and energy, strengthen kidneys, and moisten the liver, especially as a health food for pregnant women and other patients. During festival celebrations, such as the Kaimen Festival, Guanmen Festival, and Water-Sprinkling Festival, Dai people often serve colorful rice to their guests, a custom promoting the preservation of colored glutinous rice resources. During marriage ceremonies, families will often exchange a basket of home-bred glutinous rice seeds, symbolizing a bountiful and sweet life after marriage. Glutinous rice is also indispensable in Dai religious and sacrificial activities. The glutinous rice, which has been passed down and developed by the Dai ethnic group in Xishuangbanna for a long time, is closely adapted to the local ecological environment and intimately related to the Dai people's lives. It serves as one of the important material carriers of the unique cultural heritage in the local society.

## Discussion

### The diversity of rice landraces in the Xishuangbanna region

The 70 rice landraces collected in this study exhibit differences in morphology, color, and quality, reflecting the diversity of resources. The distinctive rainforest climate conditions and unique geographical environment of Xishuangbanna have fostered an ideal setting for rice cultivation, nurturing and cultivating a diverse array of rice germplasm resources. Xishuangbanna experiences a warm and humid climate throughout the year, characterized by a dry season from November to April of the following year, followed by a wet season from May to October. Correspondingly, the rice planting season is divided into early and late rice seasons [51]. The region predominantly consists of mountains and hills, encompassing approximately 95% of the area, with an extensive network of 2761 rivers coursing through it. This abundance of water resources facilitates the cultivation of upland and lowland rice, contributing to the proliferation of diverse rice landraces [23, 52].

In addition to its ecological richness, cultural diversity plays a pivotal role in fostering biodiversity. Previous studies have highlighted the intrinsic connection between the linguistic and cultural diversity of indigenous communities and local biodiversity, as these communities possess distinct yet equally effective methods of understanding and preserving biodiversity [53, 54]. For instance, the diversity of sorghum in the Kenyan mountains and maize in the Chiapas state of Mexico has been associated with local cultural and linguistic diversity [55–57]. The multi-ethnic landscape of Xishuangbanna, home to 13 native ethnic groups such as the Dai, Yao, and Hani. Each indigenous ethnic group in Xishuangbanna has a longstanding tradition of rice cultivation, relying heavily on rice production for sustenance and livelihood. These communities have developed unique rice cultures influenced by their cultural demands and environmental contexts, leading to the diversification of rice landraces [58, 59]. Examples include the Hani terraced Yuelianggu, the Bai people’s Changmaogu, and the Dai’s Haopi and Haomuxi, which were bred and domesticated to suit local ecological conditions and fulfill cultural requirements [60–62]. For instance, the Dai’s Haopi and Haomuxi are sweet, soft, and glutinous rice varieties used in religious offerings and as tribute rice presented to the emperor [63]. The Changmaogu is a rice landrace by the Bai people, adapted to high-altitude areas, high-yielding, and with high nutritional value. It is used by them for health preservation and as dowry during marriage [61].

Languages are not only just a medium of communication but also repositories of traditional knowledge [64]. The endangerment of languages often leads to the loss of traditional knowledge related to biodiversity [65, 66]. Folk naming of organisms, rooted in centuries of ethnic practices, customs, folklore, and cultural beliefs, is a vital manifestation of community language and culture [67]. It integrates economic value, utility characteristics, appearance, and growth habits of plants and animals for comprehensive identification and naming, reflecting a profound understanding by the common people. Thus, mastering these folk classifications can advance the protection of endangered languages [68]. The study on the folk naming of edible plants in the Kanekes community of Banten, Indonesia, reveals that plant names are associated with morphology, ecology, utility, and quality [69]. Similarly, a folk classification of fish by the Vaie people yields similar results [70].

Overall, the environmental conditions and traditional rice cultures of minority ethnic groups in Xishuangbanna have contributed to the domestication of unique and diverse rice varieties. These rice landraces are essential agriculture assets, serving as strategic resources for ensuring national food security and ecological stability. They serve as staple foods, nutritional supplements, and medicinal resources for indigenous communities and offer a rich reservoir of genetic material for breeding new varieties and conducting biotechnological research [2].

### Culture promotes the conservation of traditional farming varieties

Indeed, communities adapt to and shape their environments and the kinds of biodiversity that can thrive in them through their cultural practices. Traditional customary practice holds considerable sway over crop diversity in indigenous regions worldwide, including ethnic minority regions in China [71]. Apart from the Dai people, other ethnic minorities also play a vital role in preserving rice landraces diversity. For example, the Dong people cultivate Kam Sweet Rice, a local variety [72]. The preservation of local crop varieties is intertwined with the ecological environment and traditional culture of ethnic groups. For instance, the Yi people in Liangshan use buckwheat as their staple food, not only because it can be grown in cold and arid environments but also to protect Yi dietary habits, medicinal culture, festival customs, and seasonal rituals [19]; In the Sub-Saharan region, traditional knowledge has played a critical role in breeding durum wheat [73]. The rice-fish co-culture approach used in Zhejiang, China, has protected the carp diversity [74].

The rice landraces in Xishuangbanna face a rapid decline influenced by both internal and external factors, with a notable impact on the glutinous rice varieties cherished by the Dai ethnic group. This mainly includes large-scale cultivation of other cash crops, government-led cultivation of hybrid rice, impacts from foreign cultures, and inheritance vacancy [75].

Despite the potential for lower yields compared to modern hybrid varieties in certain conditions, many farmers worldwide still prefer local varieties for various reasons. Throughout millennia of agricultural history and cultural evolution, ethnic minorities have preserved, selected, and exchanged seeds within their unique cultural contexts. This ongoing process has enriched the region with diverse seeds tailored to specific needs and environmental conditions, fostering a vibrant agricultural ecosystem. Therefore, it is necessary to expedite the collection of more rice germplasm resources, safeguard the diversity of rice landraces, and concurrently preserve the rich cultural heritage of ethnic minorities.

## Conclusion

The 70 samples of rice landraces collected in Xishuangbanna display diversity across various traits such as indica or japonica type, glutinous or non-glutinous, brown rice color, grain length, and width, highlighting the abundant genetic variability present in the region. The richness in genetic diversity can be attributed to the favorable natural environment and the presence of diverse ethnic groups, both of which play pivotal roles in the conservation of rice landraces. Among the ethnic groups, the Dai people stand out as the primary cultivators of glutinous rice, showcasing higher diversity compared to other groups. Their cultural practices surrounding dietary habits, medicinal uses, festivals, and sacrificial ceremonies have contributed significantly to the preservation of glutinous rice resources, thus safeguarding their cultural heritage. However, conservation efforts face challenges due to the promotion of hybrid rice, extensive cultivation of other cash crops, the influx of external populations, and the erosion of traditional knowledge. These challenges pose unprecedented threats to rice landraces in the region. Moreover, local communities should be encouraged to continue and expand the cultivation of traditional rice varieties, thereby promoting on-farm conservation practices. This initiative not only enhances the availability of superior germplasm for rice breeding but also serves as a crucial mechanism for cultural preservation, ensuring the continuity of traditional practices and knowledge systems.

## Data Availability

All data generated or analyzed during this study were included in this published article (along with the supplementary files).

## Abbreviations

KSR: Kam Sweet Rice
JH: Jinghong
MH: Menghai
ML: Mengla
FT: First-class traits
ST: Second-class traits
